# Understanding cancer networks better to implement them more effectively: a mixed methods multi-case study

**DOI:** 10.1186/s13012-016-0404-8

**Published:** 2016-03-21

**Authors:** Dominique Tremblay, Nassera Touati, Danièle Roberge, Mylaine Breton, Geneviève Roch, Jean-Louis Denis, Bernard Candas, Danièle Francoeur

**Affiliations:** 1 Centre de recherche - Hôpital Charles-Le Moyne, Centre intégré de santé et de services sociaux de la Montérégie-Centre, 150 Place Charles-Le Moyne, J4K 0A8 Longueuil, Quebec Canada; 2 Campus de Longueuil - Université de Sherbrooke, 150 Place Charles-Le Moyne, J4K 0A8 Longueuil, Quebec Canada; 3 École nationale d’administration publique, 4750 Henri-Julien Avenue, 5th Floor, H2T 3E5 Montreal, Quebec Canada; 4 Faculty of Nursing, Université Laval, Pavillon Ferdinand-Vandry, 1050 Avenue de la Médecine, G1V 0A6 Quebec, Quebec Canada; 5 Centre de recherche du CHU de Québec - Université Laval, 11 Côte du Palais, Quebec, G1R 2J6 Quebec Canada; 6 Institut national d’excellence en santé et en services sociaux, 2535, boulevard Laurier, 5e étage, Quebec, G1V 4M3 Quebec Canada; 7 Institut national de santé publique du Québec, 190 Crémazie Blvd. East, 2nd Floor, H2P 1E2 Montreal, Quebec Canada

**Keywords:** Cancer, Case study, Governance, Health-care integration, Implementation, Mixed methods, Network

## Abstract

**Background:**

Managed cancer networks are widely promoted in national cancer control programs as an organizational form that enables integrated care as well as enhanced patient outcomes. While national programs are set by policy-makers, the detailed implementation of networks is delegated at the service delivery and institutional levels. It is likely that the capacity to ensure more integrated cancer services requires multi-level governance processes responsive to the strengths and limitations of the contexts and capable of supporting network-based working. Based on an empirical case, this study aims to analyze the implementation of a mandated cancer network, focusing on governance and health services integration as core concepts in the study.

**Methods/design:**

This nested multi-case study uses mixed methods to explore the implementation of a mandated cancer network in Quebec, a province of Canada. The case is the National Cancer Network (NCN) subdivided into three micro-cases, each defined by the geographic territory of a health and social services region. For each region, two local health services centers (LHSCs) are selected based on their differences with respect to determining characteristics. Qualitative data will be collected from various sources using three strategies: review of documents, focus groups, and semi-directed interviews with stakeholders. The qualitative data will be supplemented with a survey that will measure the degree of integration as a proxy for implementation of the NCN. A score will be constructed, and then triangulated with the qualitative data, which will have been subjected to content analysis. Qualitative, quantitative, and mixed methods data will be interpreted within and across cases in order to identify governance patterns similarities and differences and degree of integration in contexts.

**Discussion:**

This study is designed to inform decision-making to develop more effective network implementation strategies by thoroughly describing multi-level governance processes of a sample of settings that provide cancer services. Although the study focuses on the implementation of a cancer network in Quebec, the rich descriptions of multiple nested cases will generate data with a degree of generalizability for health-care systems in developed countries.

**Electronic supplementary material:**

The online version of this article (doi:10.1186/s13012-016-0404-8) contains supplementary material, which is available to authorized users.

## Background

Since the mid-1990s, networks are widely promoted as an organizational form that enables integrated care as well as enhanced patient outcomes. In broad terms, networks refer to a group of three or more organizations consciously formed, organized, and directed in ways to achieve a common goal [1, 2]. Cancer services delivery is a good example of network-based working when viewed from the perspective of service users receiving care from multiple health-care teams located in different settings [3, 4]. The seminal Calman-Hine report was the first policy document across health care to posit that cancer services should be networked, hierarchized, and integrated to allow formalizing collaboration between health-care providers and coordination across settings [5]. Since then, most national cancer control programs in developed countries take aim at solving complex problems related to care coordination (e.g., USA [6], UK [7], France [8], Australia [9], Canadian provinces [10, 11]). There has been a sustained push to reconfigure cancer services to conform to the network organizational form [12], which is becoming the rule rather than the exception [13]. However, implementing networks is a complex solution to complex fragmentation problems considering the number of actors with competing priorities, the multiple levels of governance (national, regional, local), the multiple care processes over a long period, and the various issues of the disease [14]. Nevertheless, according to Ferlie and colleagues, networks should continue to play an important role in cancer services modernization [3].

### Cancer networks as an organizational form

Being oriented toward the achievement of specific objectives, networks in the health-care sector are intended to resolve complex coordination problems involving many actors at different levels of decision-making [15, 16]. As such, a network is a form of collective action. It refers to a group of actors (clinicians, managers, governing bodies, patients) with their own goals, values, needs, representational schemes, and models of action who are often in competition and must be rallied around a common goal. By virtue of the illness and its treatments, persons with cancer receive care and services, either concurrently or at different points in time, from many different professionals and practitioners working in a variety of settings: medical clinics, hospitals, community health centers, and palliative care facilities among others. Providing coherent and continuous care requires moving from a logic based on autonomy and independency to one that is based on interdependency and the exchange of knowledge and expertise [17].

Some authors [18] have developed network typologies based on the characteristics of the links between partners (individuals or organizations). In the health-care sector, these links are described along a continuum ranging from tenuous links, such as those characterizing networks of exchange and information (e.g., communities of practice), to the more solid and durable links established by contracts among partners that characterize mandated networks (e.g., Kaiser Permanente in the USA). Between these two poles are networks characterized by links of collaboration among professionals and across organizations. The willingness to collaborate and the capacity to adapt are two necessary conditions for developing these links, which are forged over time around a shared clinical project, professional norms, and administrative requirements [19]. The experience of the United Kingdom’s cancer network has shown how a government authority that focuses on organizational restructuring and performance rather than on knowledge sharing can have an undesirable impact on the implementation and development of cancer networks [20]. While in some cases, ministry-imposed implementation of a network can be adapted so that it becomes meaningful for the professionals involved in direct service provision, in other cases, this prescriptive approach may be perceived as an intrusive control constraining professional and organizational autonomy. These observations support the idea that even mandated networks, which are the most formalized, also rely on informal and non-hierarchical links. In other words, while the form a network might take will correspond to the links that define it, it also depends on the willingness of the actors involved to collaborate with each other. Therefore, it is important to study not only the structural and organizational determinants of network implementation but also the clinical and human determinants. In this respect, current typologies are useful but could be further refined.

As for outcomes, some studies have shown the implementation of networks to be an effective method for improving health system functioning [12]. However, the conclusions vary depending on the type of network and the clientele served. Based on a review of the network integration literature, Curry and Ham [21] concluded that ministry-imposed networks were not very effective levers for improving quality of care. There is evidence demonstrating the benefits of networks, both for patients and for the health system [22–26], access to social support [27], and overall satisfaction [25]. However, researchers concur that networks’ effectiveness is extremely context-dependent and that there is no ideal network model [28].

### Governance of health-care networks

The numerous factors influencing network implementation have been well documented in the literature [12, 17]. Among them are factors related to structural characteristics (existence of a coordinating agency outside the network, coordination mechanisms and tools, size of network, internal stability), to functioning (management competence, knowledge sharing, development of capacities for innovation, mobilization of professionals, members’ efforts to participate and their engagement), and to context (health system stability, access to human and financial resources, cohesiveness, support from the community). Some studies have concluded that professional engagement, legitimacy of leaders, and trust are major determinants of a network’s performance and sustainability [17, 29, 30]. While knowledge exchange and the dissemination of innovations are presented as key components of clinical networks, these activities appear to be fostered more often through informal links than through hierarchical and formal mechanisms [18].

Governance is a multidimensional concept introduced to improve health-care quality and system effectiveness. In its simplest definition, governance refers to the coordination of collective action by a body in a position of authority [31]. “Coordination of collective action” focuses on the relationships between an organization (the National Cancer Network (NCN) in our case) and its contexts. It deals with the processes that make it possible for an organization to adapt to expectations and achieve the intended results. Provan and Kenis identified two models of network governance: shared and centralized [32]. The latter, most common in health systems, is characterized by the presence of a body that assumes the lead role because of its central position in providing services to the target clientele and its authority over the distribution of resources [32]. This body steers the strategic aspects of network implementation and development by setting up certain monitoring activities. It facilitates and coordinates the efforts of all the network partners to achieve the shared objectives.

According to Langley and colleagues [12], this lead organization can be a body within the network, such as a hospital or a health clinic, or a body outside the clinical network (e.g., a government body or regional agency). A review of the literature on the determinants of network effectiveness concluded that the existence of a coordinating body exercising external control was positively associated with networks’ capacity to achieve their objectives [12]. Provan and Kenis assert that designating a central authority in a network can have more positive consequences during a network’s emergent phase and that this effect tends to diminish as the network matures [32]. Others observed that the control exerted by a government body focused on structures and performance may have been incompatible with professional and clinical dynamics, resulting in the failure of a cancer network implementation [33].

For effective implementation in a complex organization such as a network in the health sector, some authors suggest a multi-level governance perspective [34, 35]. The “macro” level consists of a broad policy-making structure, most likely a provincial ministry of health. The “meso” level comprises a regional health board that deals with several local care delivery settings. A “micro” governance level deals with the local provider of cancer service to patients. A multi-level governance perspective takes into account the complexity of steering collective action in a network whose partners include clinicians and non-clinicians, who are both autonomous and interdependent. Multi-level governance draws attention to the policy issues as well as the challenges relevant to local health services centers (LHSCs) management and strategic practices. At each level, governance practices focus on promoting the adaptation of cancer services provision, managing relations among partners, developing knowledge through information, formulating a vision, fostering adherence to values and norms, and providing the monitoring and control needed to ensure the intended result, that is, integration, are achieved [36].

### Integration of health services

Regardless of their characteristics in governance, management, and ownership, most health-care systems face similar issues in implementing integrated networks [19]. Integration refers to both an *outcome* of implementing governance structures and a *process* that involves creating and maintaining, over time, relationships among autonomous actors that are intended to coordinate their interdependencies so they can work collaboratively to carry out a collective project (NCN in our case) [21, 37]. Integration can be examined in relation to its four dimensions: functional, clinical, professional, and normative [37]. *Functional integration* involves coherently linking together financial and information systems and network management methods. *Clinical integration* consists of ensuring that services provided by different professionals in different locations or organizations are connected over time and meet each person’s specific needs, given the knowledge available. To achieve this, clinicians agree on working methods, use shared instruments and protocols, and participate in training and knowledge exchange activities. *Professional integration* is the active participation of all professionals involved in cancer care, and more particularly, of physicians in clinical teams, on committees and in network decision-making. Lastly, *normative integration* consists of giving actors a shared value system that will help them cooperate to achieve, effectively, the collective project in which they are involved. Normative integration also allows governance to adapt to the requirements of collaboration in the network and makes professionals and organizations aware of their interdependence in providing coordinated care and services. The relative importance of each of these dimensions may vary depending on the level of governance considered. For example, macro governance at the policy level will have more influence on normative and functional integration, whereas micro-level governance will act on professional and clinical dimensions of integration. The degree of integration is determined by the extent to which providers achieve these dimensions.

### Study aim and research questions

This multi-case study aims to conduct a systematic in-depth study of a NCN implementation, with governance and services integration as the core concepts of the analysis. To achieve this objective, our analysis will answer the following questions:

1. What are the most critical contextual factors facilitating or impeding a cancer network implementation?
2. How the governance processes that support the cancer network implementation are operationalized at the health system multiple levels?
3. How are the outcomes—integration dimensions—perceived by care providers, as well as by service users (patients/families)?
4. How and why the governance processes are associated with cancer services integration?

### Implementation analysis framework

**Fig. 1:**
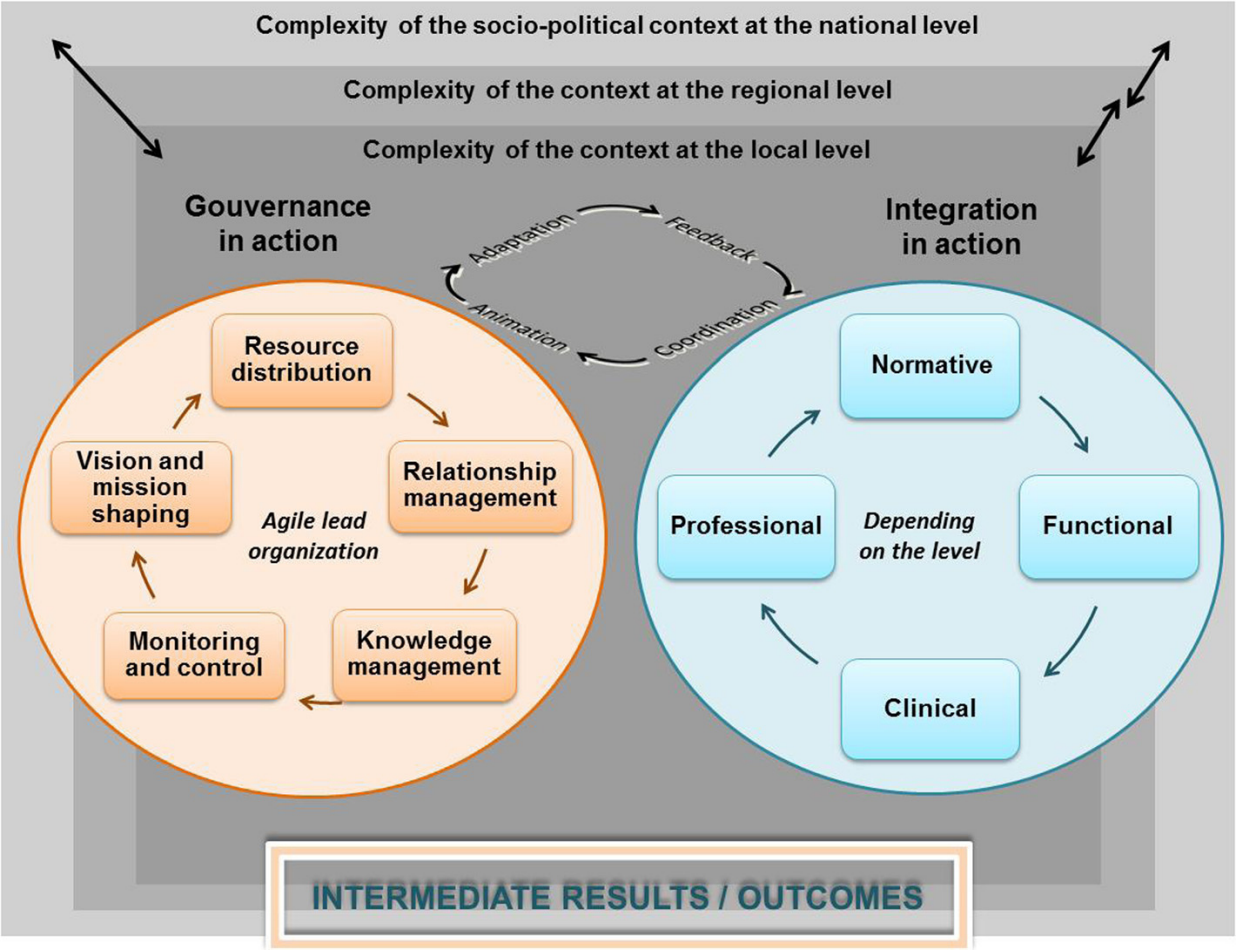
Cancer network implementation analysis framework

Figure 1 illustrates the implementation analysis framework for the study. In a summary exercise undertaken for pragmatic purposes, the proposed study is informed by a framework taking account three dimensions: issues in the implementation of a mandated network for patients with cancer, multi-level governance, and dimensions of health services integration. This framework connects the classic trilogy used to understand health systems [38], consisting of the following: (1) *contexts*—characterized by the specific features of each health system level that may influence the implantation of a NCN [12, 17]; (2) *processes*—focused on the governance practices (vision and mission shaping, resources distribution, relationship management, knowledge management, monitoring, and control) [34, 36] by a lead organization that is agile enough to adapt as the network’s implementation evolves; and (3) *outcomes*—in which the dimensions of integration (normative, functional, clinical, professional) [37] are a proxy for the cancer network implementation. This framework will guide the NCN implementation analysis in order to answer the four study questions.

### Empirical settings

The cancer network in Quebec (Canada) provides the empirical setting for the study. In 1998, the province of Quebec launched its national cancer control program (Programme Québécois de Lutte Contre le Cancer) [39]. Its main components included setting up ministry-level governance with a structure dedicated to cancer care, creating a hierarchical cancer system, integrating network-based services, promoting patient-centered interdisciplinary teamwork, and creating a nurse coordinator function, called the oncology pivot nurse [39]. The regional health authorities were responsible to operationalize the program components in the LHSCs on their geographic territories. In 2013, the policy-makers decided to further assess to what extent the network-based working produces the intended integration outcomes, namely to transcend organizational and professional boundaries and to reinforce the linkages between health-care teams, professionals, managers, and regions. The phenomenon of interest of this study, i.e., the implementation of a cancer network, is clearly prominent in this natural experiment. It represents a typical case that allows gaining explanations on how a network takes forms in the context of multi-level governance systems and to what extent intended integration results are achieved.

## Methods/design

### Design overview

The preferred design for analyzing complex interventions in natural settings such as network implementation is a nested multi-case study [40]. This design is especially appropriate to analyze the dynamics of interactions among actors in a given context [41]. In comparing cases with different characteristics, multi-case studies increase the explanatory power of the analysis of organizational processes and of the meaning actors attribute to their practices. “Nested” multi-case studies, also called case-within-case studies, examine a phenomenon of interest by subdividing it into a series of smaller cases [40]. Comparing differences and similarities in these micro-cases can shed light on variations in the phenomenon related to the different contexts in which it manifests. The proposed study also uses a sequential mixed methods approach (qualitative (dominate method) (QUAL)→quantitative (subordinate method) (quan)) in which the collection and analysis beginning with qualitative data is complemented by quantitative data [42]. Qualitative and quantitative data pertaining to the same questions serves triangulation purpose.

### Case selection

The case is a national cancer network which offers a unique opportunity to study the implementation of network-based organizational form in the “wild.” The case is subdivided into three micro-cases, each defined by the geographic territory of a health and social services region. We selected micro-cases that made up organizations in rural and urban locations, with and without specialized cancer services (e.g., academic large hospital, integrated cancer center designation), with and without the full range of cancer services (e.g., radiotherapy, ultra-specialized cancer treatments), and the time elapse since the implementation of the cancer network (Table 1). One of the micro-cases offers the potential for a longitudinal perspective on the implementation of a cancer network, given our previous studies over nearly a decade [43–46].

**Table 1:** Characteristics of the three regions (micro-cases) participating in the study

Micro-case	Geographic location	Surface (km^2^)	Population (*N*)	LHSCs (*N*)	Cancer network implementation (year)	Cancer services offered (brief description)
1	Outlying area	15,071	408,188	5	2013	No supra regional team Radiotherapy outside the region
2	Metropolitan area	498	1,981,672	12	2014	Full range
3	Rural/semi-urban/urban area	11,111	1,470,252	11	2001	Breast cancer supra regional team Radiotherapy introduced in 2013

*LHSCs* local health services centers

For each micro-case, two LHSCs will be selected based on their differences in terms of certain determining characteristics: scope of the LHSC’s cancer mandate (local or regional); degree of specialization (community-based/general, specialized, or ultra-specialized); and cancer clientele habits of cancer services utilization. The six LHSCs will be selected in partnership with the decision-makers having intimate knowledge of these organizations. The formal partnership structure of the study is presented in Additional file 1.

### Qualitative data collection

The qualitative data will be generated from four sources using different methods. We will begin by reviewing pertinent documents (e.g., minutes of meetings, action plans, regional and governmental reports). Homogeneous focus groups (FGs) will be conducted with three groups of actors from each micro-case: regional-level decision-makers, service users, care providers, and front-line managers from each LHSC. Each group will consist of 8–10 participants [47] selected through a purposive snowball sampling [48]. The FGs with care providers will explore the governance processes, the role of governance in achieving outcomes for each dimension of integration, and the processes of coordination and adaptation to network-based working. The FGs with service users will focus on describing integration based on patients’ and families’ perceptions of their care experiences.

Semi-directed interviews will be conducted with key informants to explore more deeply and validate the various aspects raised in the FGs. Representatives of various government authorities and agencies will be recruited through a purposeful sampling approach [48]. Based on our previous studies, we estimate that around 30 interviews will be required per region and another dozen at the provincial level in various ministries, such that a total of about 100 interviews will be conducted. This number will be adjusted depending of the type and depth of information collected and in accordance with the principle of data saturation [49]. We will use systematic data collection grids for FGs and interviews, based on the concepts in the conceptual framework developed for the study. The grids will be adjusted to suit the type of information being sought and the persons providing the information.

### Qualitative data analysis

Qualitative data from document review, focus groups, and semi-structured interviews will be analyzed in QDA Miner using a thematic coding system developed from our conceptual framework [50]. First, each case will be analyzed separately. Then, a cross-case analysis will be done to compare differences and similarities and to identify patterns in the governance processes, the extent of integration, and the results revealed by the analyses.

### Quantitative data collection

The quantitative data will be collected through a survey of stakeholders (clinicians, executive directors, managers). A list of potential respondents will be developed with the partners of the study. Given the number of people involved in providing services to persons with cancer, we estimate there will be around 100 participants per LHSC. Participants will be invited to respond to an electronic questionnaire using the *LimeSurvey* platform. Based on our previous studies, we expect a response rate of 60 % using systematic reminders at 2 and 4 weeks by email [51].

The level of integration will be measured using an instrument developed by Denis and colleagues [52, 53] in a study on the creation of health and social services centers (HSSCs) and the development of integrated health services networks. This instrument was inspired by the questionnaire originally developed by Shortell and colleagues [54, 55] for the Health System Integration Study and later adapted by Dobrow and colleagues [56] to evaluate the dimensions of cancer network integration in the province of Ontario (Canada). The instrument consists of 64 questions evaluated on a five-point Likert-type scale ordered to reflect progression in the degree of implementation of the four dimensions of integration. Participants are asked to respond to the questions based on their perceptions of the activities at different levels (e.g., *To what extent are the managers of your local network involved in the quality management activities for cancer services? To what extent are the managers of your regional network involved in the quality management activities for cancer services?*) The questionnaires are tailored for different types of respondents (clinicians, executive directors, managers) and include sociodemographic characteristics (e.g., age, sex, work setting, function).

To reinforce its validity, the questionnaire contains two vignettes presenting complex care trajectories requiring reciprocal coordination among professionals and among organizations. For the proposed study, we will adapt a vignette used in a previous study by our team to evaluate the implementation of a regional cancer network [45, 57]. The vignette illustrates the trajectory of a patient with colon cancer from diagnosis through active treatment and into the palliative phase.

### Quantitative data analysis

The data collected through the questionnaires will be analyzed using descriptive statistics. We will construct a score for level of integration by grouping individual responses and based on an index of the agreement on this question among respondents of the same LHSC. Scores for each dimension of integration will be constructed in five stages following the detailed procedure developed by James, Demaree, and Wolf [58]. In the end, these scores will range from 0 to 10, with scores of 5 and under considered weak, 6 average, 7 good, 8 excellent, and 9 or above exceptional. Two members of our team (JLD and MB) have already carried out this exercise of constructing an integration score in the study on HSSCs. The quantitative analysis results will be integrated with those of the qualitative analysis. The final step will consist of comparing the micro-cases and performing a cross-case analysis to document the degree of cancer network implementation.

### Study validity

The quality and validity of the study will be key concerns throughout the research process. To address these, we will use the framework and criteria of Mays and colleagues [58], which include the following: appropriate use of a variety of methodological approaches, data triangulation, triangulation of the views of researchers and potential users, use of an analytic framework to guide data collection and analysis, collaborative sampling of cases and selection of participants, and systematic approach to data collection and analysis [59]. These approaches will strengthen the study’s internal validity (trustworthiness of explanatory significance). External validity (capacity to generalize results) will be ensured by including different micro-cases and by analyzing how multi-level governance and their particular contexts influence the implementation of their network. This will allow us to identify the conditions required to generalize the results with other LHSNs and regional networks [60] and to maximize potential applicability and transferability of findings to cancer networks in other developed countries.

### Knowledge transfer plan

The proposed study focuses on utilization, in that the resulting knowledge should align with the decision-making challenges of the potential users of the study’s results [61]. Consequently, throughout the study, we will use an integrated knowledge transfer (IKT) approach, in which information will be shared throughout the implementation analysis process to foster interactions among actors at different levels of decision-making, in order to improve communication and reinforce learning [62]. An IKT is the result of a collaborative process and promotes knowledge application at all stages. The framework proposed by Baumbusch and colleagues allows us to conceive IKT as a bidirectional relationship between researchers and knowledge users, in which each participates in the co-construction of knowledge though a collaborative approach [63]. That framework proposes two core concepts: content (the knowledge in itself) and process (application of the knowledge). Within this framework, our project involved consolidating the questions arising from the concerns expressed by policy-makers, and our aim is to create and share the emerging results in real time through research activities geared toward questions expressed by the potential knowledge users. In this way, new knowledge will be constructed that responds to their needs. The research results can thus be applied at the opportune moment, that is, when decisions need to be taken—whether at the local, regional, or provincial level—to optimize the integration required to operationalize the cancer network. Within a formal partnership structure (see Additional file 1), knowledge users at different levels of decision-making in the health system will be involved in interpreting results and in formulating concrete avenues of intervention to support decisions aimed at optimizing the integration of practices and cancer services.

### Study status

The approval of the Research Ethics Board of the Research Center of the Charles-Le Moyne Hospital (HCLM) was obtained for all study procedures in participating local health services centers (record number: MP-HCLM-14-010). Recruitment and data collection for this study began in July 2014 and will continue for the next year.

## Discussion

The expected benefits of the research project are numerous and specific depending on the time frame of the study. First, the case study will produce new data on the achievement of the integration outcomes envisioned in the national cancer control program regarding the NCN implementation, as well as on the unintended or undesirable outcomes of the implementation of network-based working. Second, it will be possible to better understand how the multi-level governance contributes to cancer services integration and how the contexts influenced this relationship. Third, the in-depth analysis of transformations in practices introduced by the NCN will help explain the gaps between the intended results and the results as they occurred in natural settings. Fourth, the strategies taken to ensure the validity of the study will help generate data and knowledge to guide decision-making and to deal more effectively with the challenges of implementing the cancer network.

## Additional file

Additional file 1: Partnership structure of the study. Partnership structure for integrated knowledge transfer. (DOCX 61 kb)

## Abbreviations

FG: focus group
HSSC: health and social services center
IKT: integrated knowledge transfer
LHSC: local health services center
NCN: National Cancer Network
QUAL: qualitative (dominate method)
quan: quantitative (subordinate method)
