# Supplementary material for: Understanding cancer networks better to implement them more effectively: a mixed methods multi-case study
Source: Implement Sci. 2016 Mar 21;11:39. doi: 10.1186/s13012-016-0404-8 (PMC4802906; doi:10.1186/s13012-016-0404-8)
Supplement: Additional file 1: — Partnership structure of the study. Partnership structure for integrated knowledge transfer. (DOCX 61 kb) [file 13012_2016_404_MOESM1_ESM.docx]

# **Additional file 1: Description of the partnership structure of the study**

## Figure 1 Partnership structure for integrated knowledge transfer

The partnership structure of the study illustrated in Figure 1 was discussed with key persons knowledgeable of the cancer network implementation in their region. The macro level is expected to include representatives of the governing body at the policy level. Discussions between the research team and regional key informants led to consensus around two priorities. The first is that formal links need to be established among actors at different decision-making levels. Hence, the planned partnership structure is centred around applying knowledge through a process of resolving problems [[1](#_ENREF_1)] identified over the course of implementing the cancer network. The second is that the structure needs to be minimalist, flexible, and integrated into existing management structures. Formalizing the structure should enable the integrated knowledge transfer (IKT) to be operationalized, but it would be important to avoid creating parallel committees that consume time and energy and make it likely that the structure would collapse after the research team has completed its mandate. The integrated structure will enable users to apply knowledge independently. We believe it is realistic to expect, at a minimum, that the strategic level of the partnership would meet once before each of the interim reports at 12, 24, and 26 months, and again six months after the end of the project. With regard to the management level, it was agreed that a core group would be created, consisting of the three regional managers and the three co-managers, who will meet with certain members of the research team every six weeks. Around this core group will be a number of key persons who will become involved as needed as the work progresses. The core group is expected to play a pivotal role in knowledge transfer and the dissemination of best network practices and to continue functioning after the project is completed. As the project unfolds, it will be important to identify other stakeholders with whom partnerships should be established. The mandate, objectives, and roles of the partners at each level will be developed with the collaborators, in response to their needs, and will be centred around an “action imperative” and the receptive capacity of the organizations and people in each setting [[2](#_ENREF_2)]. As such, the degree of partnership developed between the research team and the study’s collaborators will depend on a variety of external factors, and will range from minimal interaction involving the passive transmission and dissemination of information, all the way to active collaboration involving shared decisions on the implementation analysis process and the conclusions to be drawn from the findings [[3](#_ENREF_3)].

**References**:

1. Denis JL, Lemieux-Charles L, Champagne F: **A knowledge utilization perspective on fine-tuning dissemination and contextualizing knowledge.** In *Using knowledge and evidence in health care.* Edited by L. Lemieux-Charles, F. Champagne: University of Toronto; 2004: 18-40

2. Denis J-L, Lehoux P, Tré G: **L'utilisation des connaissances produites.** In *Approches et pratiques en évaluation de programme.* Edited by V. Ridde, Christian Dagenais. Montréal: Les Presses de l'Université de Montréal; 2009

3. Patton MQ, LaBossière F: **L'évaluation axée sur l'utilisation.** In *Approches et pratiques en évaluation de programme.* Edited by Valéry Ridde, Christian Dagenais. Montréal: Les Presses de l'Université de Montréal; 2009.
